# Levels of angiotensin-converting enzyme 1 and 2 in serum and urine of children with Sickle Cell Disease

**DOI:** 10.1590/2175-8239-JBN-2020-0174

**Published:** 2021-05-03

**Authors:** Ho Chi Hsien, Dulce Elena Casarini, João Tomas de Abreu Carvalhaes, Fernanda Aparecida Ronchi, Lilian Caroline Gonçalves de Oliveira, Josefina Aparecida Pellegrini Braga

**Affiliations:** 1 Universidade Federal de São Paulo, Escola Paulista de Medicina, Departamento de Pediatria, Disciplina de Nefrologia, São Paulo, SP, Brasil.; 2 Universidade Federal de São Paulo, Escola Paulista de Medicina, Departamento de Medicina, Disciplina de Nefrologia, São Paulo, SP, Brasil.; 3 Universidade Federal de São Paulo, Escola Paulista de Medicina, Departamento de Pediatria, Disciplina de Hematologia, São Paulo, SP, Brasil.

**Keywords:** Anemia, Peptidyl-Dipeptidase A, Kidney, Glomerular Filtration Rate, Anemia, Peptidil Dipeptidase A, Rim, Taxa de Filtração Glomerular

## Abstract

**Introduction:**

Sickle cell nephropathy begins in childhood and presents early increases in glomerular filtration, which, over the long term, can lead to chronic renal failure. Several diseases have increased circulating and urinary angiotensin-converting enzyme (ACE) activity, but there is little information about changes in ACEs activity in children with sickle cell disease (SCD).

**Objective:**

We examined circulating and urinary ACE 1 activity in children with SCD.

**Methods:**

This cross-sectional study compared children who were carriers of SCD with children who comprised a control group (CG). Serum and urinary activities of ACE were evaluated, as were biochemical factors, urinary album/creatinine rates, and estimated glomerular filtration rate.

**Results:**

Urinary ACE activity was significantly higher in patients with SCD than in healthy children (median 0.01; range 0.00-0.07 vs median 0.00; range 0.00-0.01 mU/mL·creatinine, p < 0.001. No significant difference in serum ACE activities between the SCD and CG groups was observed (median 32.25; range 16.2-59.3 vs median 40.9; range 18.0-53.4) mU/m`L·creatinine, p < 0.05.

**Conclusion:**

Our data revealed a high urinary ACE 1 activity, different than plasmatic level, in SCD patients suggesting a dissociation between the intrarenal and systemic RAAS. The increase of urinary ACE 1 activity in SCD patients suggests higher levels of Ang II with a predominance of classical RAAS axis, that can induce kidney damage.

## Resumo

**Introdução:**

A nefropatia falciforme começa na infância e apresenta aumentos precoces na filtração glomerular, que, em longo prazo, podem levar à insuficiência renal crônica. Várias doenças têm aumentado a atividade da enzima conversora da angiotensina (ECA) urinária e circulante, mas há pouca informação sobre alterações na atividade das ECAs em crianças com doença falciforme (DF).

**Objetivo:**

Examinamos a atividade da ECA-1 circulante e urinária em crianças com DF.

**Métodos:**

Este estudo transversal comparou crianças que eram portadoras de DF com crianças que compunham um Grupo Controle (GC). As atividades séricas e urinárias da ECA foram avaliadas, assim como os fatores bioquímicos, a relação albumina/creatinina urinária e a taxa de filtração glomerular estimada.

**Resultados:**

A atividade urinária da ECA foi significativamente maior em pacientes com DF do que em crianças saudáveis (mediana 0,01; intervalo 0,00-0,07 vs mediana 0,00; intervalo 0,00-0,01 mU/mL·creatinina, p < 0,001. Não foi observada diferença significativa nas atividades séricas da ECA entre os grupos DF e GC (mediana 32,25; intervalo 16,2-59,3 vs mediana 40,9; intervalo 18,0-53,4) mU/mL·creatinina, p < 0,05.

**Conclusão:**

Nossos dados revelaram uma alta atividade urinária da ECA-1, diferente do nível plasmático, em pacientes com DF, sugerindo uma dissociação entre o Sistema Renina Angiotensina Aldosterona (SRAA) intra-renal e sistêmico. O aumento da atividade urinária da ECA-1 em pacientes com DF sugere níveis mais elevados de Ang II com predominância do eixo clássico do SRAA, que pode induzir lesão renal.

## Introduction

Sickle cell disease (SCD) is characterized by vaso-occlusive crisis (VOC) and endothelial damage that determine chronic and progressive damage to organs, including the kidneys^1^^.^ The most common renal complications include asymptomatic gross hematuria, hyposthenuria, necrosis of the renal papilla, greater glomerular hyperfiltration, and proteinuria.^2^ Early diagnosis in childhood is very important so that preventive measures and monitoring can be practiced, thereby preventing kidney failure in adulthood^3^. Glomerular damage, although less frequent, leads to progressive loss of kidney function, culminating in chronic kidney failure in approximately 20% of patients^4^.

In a multicenter study, the glomerular filtration rate was found to increase in infants who experienced onset of SCD at nine months of age^5^^.^ Hyperfiltration is a risk factor for developing proteinuria and chronic kidney disease in SCD.^6^ These renal changes may be accompanied by changes in the renin angiotensin aldosterone system (RAAS), and studies have described how SCD patients experience decreases in microalbuminuria and proteinuria with the use of angiotensin I-converting enzyme (ACE1) inhibitors^7^. Recently, Thrower et al. (2019) demonstrated that patients with proteinuria who received RAAS blockade presented delayed loss of kidney function in patients with SCD^8^.

The imbalance of classical ACE 1/Angiotensin II (Ang II) /AT1 receptor axis and counter-regulatory ACE 2/Ang 1-7/MAS receptor axis was studied by Belisario et al. (2018) highlighting that ACE 2 and Ang 1-7 were reduced in pediatric SCD with increase of ACE 1 and Ang II, inducing kidney damage^9^.

ACE 2 is a type I integral membrane protein that shares 42% homology with ACE 1 and contains a single zinc dependent catalytic site able to cleave the vasoconstrictor Ang II to the vasodilator Ang 1-7^10^^,^ 11. This enzyme is found in many tissues and is expressed in the kidney, especially in mesangial, proximal, and collecting duct cells. ACE 1 inhibitors are not able to block ACE 2^12^^,^ 13.

The changes in the levels of enzymes and peptides from RAAS using SCD animal models were described by Roy et al. (2018). Their findings suggest that blockade of AT1 receptor, together with agonism of AT2R signaling, prevents sickle glomerulopathy^14^. Several authors have described a statistically significant increase of ACE 1 activity in patients with chronic renal disease^15^^,^ 16, type 1 diabetes, and systemic inflammatory disease involving kidney impairment before starting enzymatic treatments, as described by our working group^17^.

Studies of serum and urinary ACE 1 activities in SCD, especially in children, are limited. The present study aimed to evaluate the modulation of serum and urinary ACE 1 and ACE 2 activities in pediatric SCD, important for understanding the role of these enzymes in the sickle cell nephropathy.

## Methods

### Compliance with ethical standards

This study was approved by the Research Ethics Committee of the Federal University of São Paulo (UNIFESP) number 0557/02. All parents or guardians of the participants were given information about the study. After clarifying doubts about the study, these parents or guardians were asked to sign an informed consent form before the study began.

### Subjects and sample collection

This single-center, descriptive, cross-sectional study included patients with SCD who were monitored in the pediatric clinic of a reference hospital. We also recruited healthy volunteers of a similar age from a private elementary school in southeastern São Paulo (Brazil), with no recent history of disease or medication use, who served as the Control Group.

A standardized form was used to record data from participants, such as weight, height, blood pressure (BP), numbers of VOC/year of age, calculated using the total number of occurrences of VOCs per patient per year divided by the patient's age in years (number of events/age of the patient in years). The z scores for the anthropometric ratios weight/age, height/age, and weight/height were used to evaluate nutritional status. Using the World Health Organization growth charts, nutritional status was classified using body mass index.^18^ Three BP measurements were taken by the researcher himself and classified according to The Fourth Report on the Diagnosis, Evaluation, and Treatment of High Blood Pressure in Children and Adolescents^19^.

### Exclusion Criteria

Patients or controls using corticosteroids, nonsteroidal anti-inflammatory medications, anticonvulsants, antihistamines, bronchodilators, digitalis, or hypotensive drugs were excluded. Individuals with a VOC in the preceding three months or fever during sample collection or blood transfusion in the month preceding recruitment were also excluded. No participant was a current user of hydroxyurea, iron chelation, or renal replacement therapy.

### Sample collection and analytical methods

Urinary samples were collected from volunteers in presence of inhibitor cocktail, EDTA-free, and preserved at -20ºC until processing.

The urine used for ACE 1 activity measurement was concentrated to 1 mL using a centricon centrifugal filter 30 kDa cutoff (Millipore, Billerica, MA) and dialyzed in the same filter against 50 mmol/L Tris-HCl, pH 8.0, with 150 mmol/L NaCl.

Angiotensin I-converting enzyme catalytic activity was determined fluorimetrically as described by Friedland and Silverstein^20^. An aliquot of serum (10 µL) and urine prepared as describe above (50 µL) was incubated with a 200 µL assay buffer (solution containing 1 mmol/L Z-Phe-His-Leu (Z-FHL) in 100 mmol/L sodium borohydride buffer, pH 8.3, 300 mmol/L NaCl, and 0.1 mmol/L ZnSO_4_) for 10 min at 37°C. The enzymatic reaction was stopped by the addition of NaOH (0.28 N; 1.5 mL) and incubated with o-phthaldialdehyde (20 mg/mL methanol; 100 µL; 10 min). The fluorescence reaction was stopped by the addition of HCl (3 N; 200 µL). The dipeptide His-Leu thus released was measured fluorometrically (λ_ex_: 360 nm; λ_em_: 465 nm) using the F-200 fluorimeter (Infinite Model; Tecan; Grödig, Austria). Calculation was based on a standard curve, then values were normalized by the creatinine concentration for urinary samples.

ACE 2 activity was also determined by fluorimetry, using the substrate MCA- APK-Dnp (30 mM, λ_ex_: 320 nm; λ_em_: 420 nm). Buffer (Tris-HCl 50 mM, NaCl 1 M, ZnCl2 10 mM, captopril 10 mM, pH 6.5) and serum samples (10 µL) were pre-incubated for 30 min in the presence or absence of ACE 2 inhibitor (DX600, 20 mM). Substrate was added and the reactions were measured for 60 min by F-200 fluorimeter (Infinite Model; Tecan; Grödig, Austria). Arbitrary units of fluorescence were registered, calculations were based on a fluorescence standard curve (OMNIMMP), and the time point 0 was used as internal blank.

Urinary albumin excretion was measured using a turbidimetric immunoassay and was expressed as the albumin/creatinine concentration ratio (UACR). Elevated albuminuria was defined as UACR > 30 mg/g^21^. Urinary creatinine was measured using a modified Jaffé reaction and an automated analyzer by Hitachi 912 - Roche^22^.

To obtain the estimated glomerular filtration rate (eGFR), the updated Schwartz bedside equation was used. Hyperfiltration was defined as eGFR ≥150 mL/min/1.73 m^2^ 23.

### Statistical analysis

Based on our preclinical studies of ACEs activity in 30 sickle cell children, with power of 0.8 and α<0.05, a sample size of 28 was calculated.

Normally distributed continuous variables (weight, body mass index (BMI) and height Z scores, and SBP) were analyzed quantitatively and expressed as mean values ± standard deviation (SD), and the three-sample Anova-test was used to determine the statistical significance of the mean values and the categorical data.

The non-normally distributed data are expressed as median values and inter-quartile range (IQR) and non-parametric tests were performed. Baseline parameters in the SCD group vs the Control Group were compared using the Mann-Whitney U-test and the Kruskal-Wallis test.

Spearman's (rho) correlation coefficient was used to determine associations between outcomes. A two-tailed p-value less than 0.05 was considered to be statistically significant.

## Results

### Anthropometric and bp parameters

Anthropometric, cardiovascular, and biochemical parameters of the participants are shown in Table 1. The SCD group included 32 children without VOC in sample collection moments. Ten (31.2 %) of these had VOC with an average of 0.4 (0.11 - 0.76) episode per year old. The Control Group had 22 children.

**Table 1:** Anthropometry, blood pressure, biochemical markers, serum, and urinary angiotensin-converting enzymes 1 (ACE 1) and 2 (ACE 2) activities in children with sickle cell disease and control group

	Sickle Cell Disease	Control Group	p
Sex (M/F)	15/ 17	13/9	
Age (years)	10.74 (3.55)	11.98(1.75)	0.002
Weight (kg)	31.60 (11.70)	48.00 (15.10)	<0.001
BMI	15.95 (14.67 - 18.37)	18.95 (18.17 - 24.95)	<0.001ϯ
BMI z-score	-0.62 (1.42)	0.96 (1.08)	<0.001
Height z-score	-0.90 (1.40)	-0.05 (1.03)	0.026
Diastolic blood pressure	63.70 (10.20)	70.90 (6.70)	0.003
Systolic blood pressure	97.70 (13.3)	106.10 (10.20)	0.028
Serum creatinine (mg/dL)	0.39 (0.13)	0.55 (0.08)	<0.001
UACR (mg/g Cr)	10.21 (5.56-19.06)	24.49 (3.37 -25.79)	0.58 ϯ
Estimated glomerular filtration rate (mL/min/1.73 m2)	150.8 (36.10)	113.4 (14.8)	<0.001
Serum ACE1 activity (nmol/mL/min)	32.35 (22.52 - 38,97)	33.29 (33.29 - 37.57)	0.066 ϯ
Serum ACE 2 activity (nmol/mL/min)	88.85 (3.99 - 149.84)	91.77 (57.22 - 158.08)	0.058
ACE 1/ ACE 2 activity ratio	0.59 (1.31)	0.40 (0.12)	0.157
Urinary ACE 1 activity (nmol/min/mg Cr)	0.10 (0.00- 0.16)	0.00 (0.00 - 0.01)	<0.002 ϯ

Data presented as mean ± SD, median (IQ 25-75). Student's t-test ; ϯ Mann-Whitney test. BMI: Body mass index; UACR: urinary albumin to urinary creatinine concentration ratio. ACE 1: Angiotensin I-converting enzyme / ACE 2: Angiotensin II -converting enzyme

The parameters weight, BMI, Z-score of BMI and height, DBP (diastolic blood pressure) and serum creatinine in the Control Group were significantly higher than those in the SDC group (p < 0.05). We observed that eGFR in the SCD group was significantly higher than the Control Group (p <0.001). There were no significant differences in the UACR between the SCD group and the Control Group (table 1).

**Figure 1:**
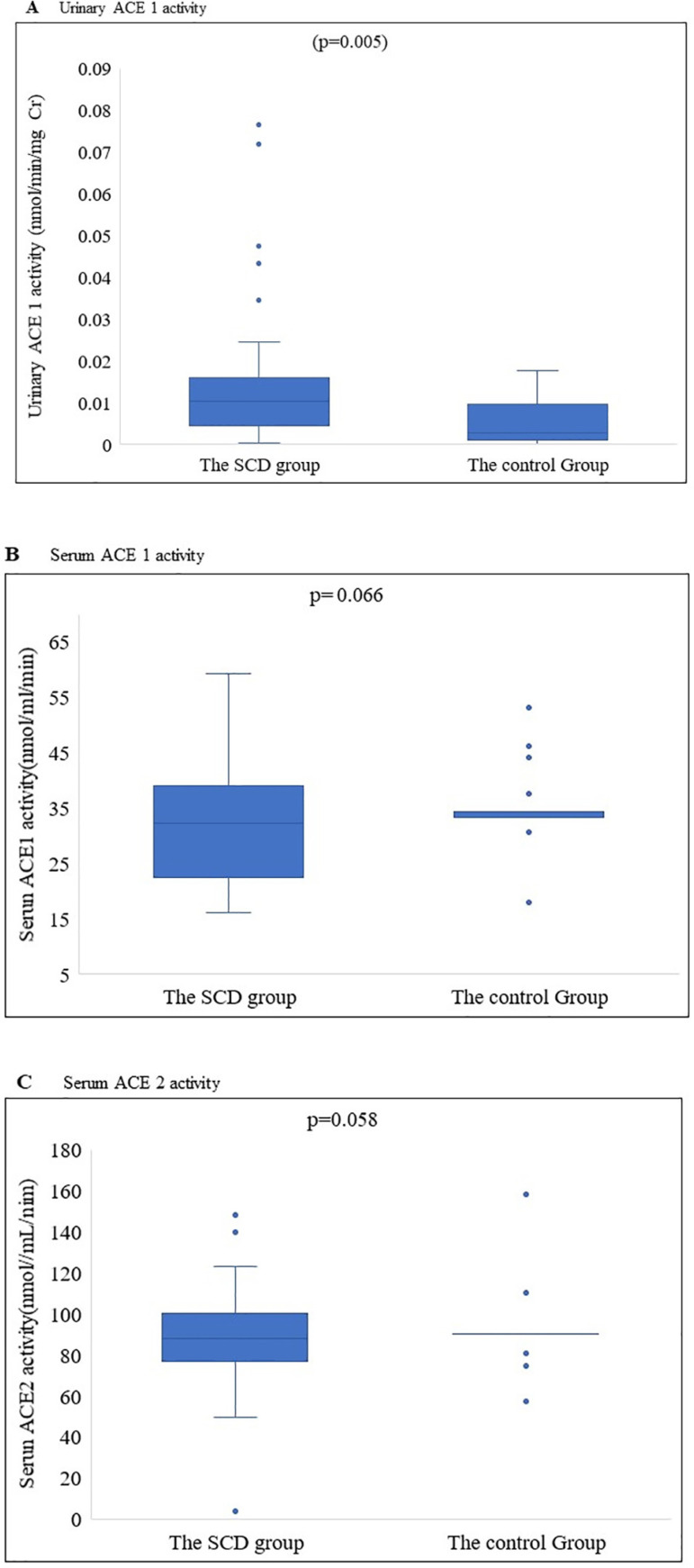
Activity of urinary ACE 1 (A), serum ACE 1 (B), and serum ACE 2 (C) in sickle cell group. Significant difference was found in urinary ACE 1 activity in the SCD group when compared with the Control Group (p=0.005) (A); the SCD group and the Control Group had similar serum ACE 1 and ACE 2 activities (p= 0.066; 0.058) (B/C).

We observed lower urinary ACE 1 activity in the SCD group versus control (p=0.005), and the SCD group and the Control Group had similar serum ACE 1 and ACE 2 activities (p 0.066; 0.058) (Table 1 and Figure 1).

**Figure 2:**
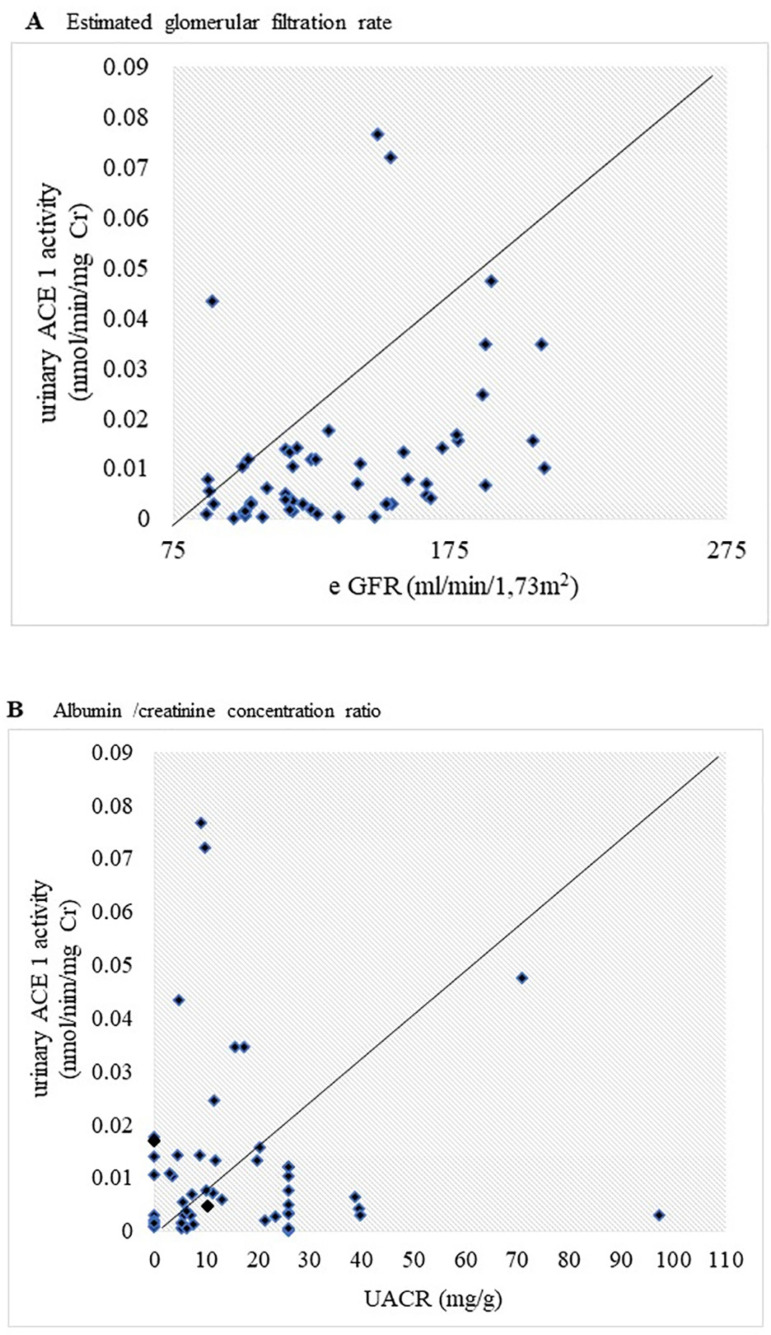
Correlations between urinary ACE 1 activity, estimated glomerular filtration rate (eGFR), and albumin/creatinine concentration ratio (UACR) in sickle cell disease: (A) Estimated glomerular filtration rate in sickle cell disease. (B) Albumin/creatinine concentration ratio. Spearman's (rho) correlation coefficient is shown. Statistically significant differences were established at p < 0.05. Urinary ACE 1 activity was positively correlate with eGFR (rho=0.388, p=0.028) (R=0,339) but not correlated with UACR (rho= -0.058, p=0.751).

For the SCD group the values of urinary ACE 1 activity exhibited a correlation with eGFR (rho=0.388, p=0.028) (regression, R =0.339) and no correlation with UACR (rho= -0.058, p=0. 0.751) (figure 2). Systolic BP (SBP) and diastolic BP (DBP) were not correlated with serum ACE 1 (rho=-0.57, p=0.680; rho=-0.175, p=0.205) or urinary ACE 1 activity (rho=0.148, p=0.418 and (rho=0.152, p=0.406) respectively.

### Serum ACE 1, ACE 2, ACE 1 / ACE 2 ratio and urinary ACE 1 activities in SCD with VOC.

No significant differences were observed between the urinary ACE 1 activity tertiles when the SCD >0.4 VOC/year old and SCD <0.4 VOC/year old groups were compared (p=0.535) (table 2).

**Table 2:** Anthropometry, blood pressure, laboratory markers, serum, and urinary angiotensin converting enzyme (ACE) activity in children with sickle cell disease

		Sickle Cell Disease	
	**<0.4 Vaso-occlusive crises/year old**	**> 0.4 Vaso-occlusive crises/year old**	**p**
Variables			
Age	10.47 (3.65)	11.35 (3.44)	0.988
Weight (Kg)	26.25 (23.35-36.55)	29.0 (24.2-36.37)	0.617 ϯ
BMI	15.95 (14.57-17.77)	17.15 (14.82-19.72)	0,889 ϯ
z-score BMI	-0.52 (0.96)	-0.84 (2.15)	0,027
z-score height	-0.46 (-1.73-0.22)	-1.38 (-1.90- 0.41)	0.029 ϯ
Diastolic blood pressure (mm Hg)	65.75 (59.25-70.75)	57.75 (55.0-70.62)	0. 516 ϯ
Systolic blood pressure (mm Hg)	96.25 (14.71)	100.80 (9.40)	0.036
Serum creatinine (mg/dl)	0.40 (0.32-0.45)	0.31 (0.29-0.33)	0.18 ϯ
Estimating glomerular filtration rate (mL/min/1.73m2)	145.55 (113.85-166.37)	182.21 (153.57-192.12)	0.009 ϯ
UACR (mg/g Cr)	9.31 (5.58 - 14.06)	13.48 (2.66 -53.26)	0.411 ϯ
Serum ACE 1 (mU/mL)	31.85 (22.57 - 38.72)	32.64 (20.52 - 39.70)	0.795 ϯ
Serum ACE 2 (mU/mL)	88.95 (64.35-100.46)	86.66 (78.65-113.57)	0.704 ϯ
ACE 1/ACE 2 Ratio	0.33(0.27-0.46)	0.34 (0.21-0.43)	0.366 ϯ
Urinary ACE 1 (nmol/min/mg Cr)	0.0073 (0.0040 - 0,0192)	0.0130 (0.0056 - 0.0187)	0.535 ϯ

Data presented as mean ± SD, median (IQ 25-75). Student's t-test. ϯ Independent samples Kruskal-Wallis test. Abbreviations: BMI: body mass index, UACR: urinary albumin to urinary creatinine concentration ratio, ACE 1: Angiotensin I-converting enzyme, ACE 2: Angiotensin II-converting enzyme.

## Discussion

Currently, methods to identify young SCD patients with the highest risk of developing renal complications remain limited to microalbuminuria and creatinine analysis. It is therefore important to develop prognostic biomarkers for these complications, so these patients can be identified and thereby targeted for earlier preventative therapies. However, the physiological factors that promote these early preclinical changes in urinary albumin excretion and hyperfiltration remain unclear.

Our first novel observation in this cohort of pediatric patients with SCD was the significantly higher levels of urinary ACE 1 activity compared with our Control Group (Figure 1). The SCD exhibits perfusion paradox that is characterized by hypoperfusion in microcirculatory beds occluded by hemoglobin S-containing erythrocytes while hyper perfusion in the systemic (macro) circulation and a number of regional vascular circuits. Cortical hyper perfusion and medullary hypoperfusion occurs in the kidney, and the medullary ischemia stimulates release of vasoactive mediators, resulting in glomerular hyperfiltration^24^.

We found higher urinary ACE 1 activity in the SCD group and a significant positive correlation with glomerular hyperfiltration. The evolution of sickle cell nephropathy has been compared to type I diabetes nephropathy with renal hyperfiltration^24^^,^ 25. We have demonstrated increased filtration rates of 150.8 mL/min/1.73m^2^ among individuals with SCD, similar to what has been observed in diabetic nephropathy^26^.

Our patients had no microalbuminuria and urinary ACE 1 activity was not correlated with UACR (Figure 2). These results differ from those described by Hallab et al. (1992) and Burns et al. (2017), in which urinary ACE 1 activity was elevated in type 1 diabetic subjects, especially in patients with microalbuminuria, suggesting an early indication of lesions in vascular endothelial cells^27^^,^ 28. In addition, Belisario et al. (2019) described that SCD children with persistent albuminuria (PA) also presented increased urinary levels of ACE 1^9^.

Casarini et al. (2001) described a correlation between urinary ACE 1 and BP suggesting that the somatic and N-domain urinary ACE 1 are produced locally and released by the tubular cells in normal conditions and in response to ischemic kidney damage^29^.

In our second observation, serum ACE 1 and 2 activities were found to be similar in the SCD group and the Control Group. Regarding ACE 1 activity, there was divergence in the findings. Bennion et al. (2016) described that in patients with ischemic stroke, serum levels of ACE 1 activity were not lower than the control group. Immediately after stroke, they were significantly decreased by nearly 15% compared to acute levels at three days after stroke^30^^.^ In chronic kidney disease stage 3-5 patients, without previous history of cardiovascular disease, circulating ACE 1 activity was significantly higher^31^.

Studies reported increased serum ACE 2 activity in vascular disease, for example, in patients with significant obstructive coronary artery disease, ischemic stroke or type 1 diabetic, with microvascular or macrovascular diseases^30^^,^ 32. In our study, the patients with SCD showed a tendency for higher serum ACE 2 activity, although there was no statistical difference in relation to the Control Group. We can attribute the finding to the patients' not having had a VOC or complications due to this process.

We found that serum ACE 1 activity was lower in individuals with SCD than Control Group, but not significantly (p<0.066). In our study, no correlation was observed between serum ACE 1 activity and BP. This finding is not in accordance with the results described by Franco et al. and Landazuri et al.^33^^,^ 34 These studies demonstrated correlations between these parameters in healthy children. This finding may be attributed to the fact that children with SCD have endothelial injury. As previously reported in studies of SCD, we noted that BP levels were lower in the SCD group than in the Control Group^34^^-^ 36.

Our findings are similar to the conclusions of Febba et al. (2009) and Burns et al. (2017)^28^^,^ 37. These studies also did not find a correlation between urinary ACE 1 activity and SBP or DBP values. According to a review of studies of renal ACE 1 activity and BP conducted by Bernstein et al. (2013)^37^ certain experimental results linked urinary ACE 1 activity with high BP; however, additional studies are necessary to improve our understanding of this correlation.

Our results indicated increased urinary ACE 1 activity, which may reflect intrarenal RAAS activation, potentially leading to effects on albumin excretion. We did not find correlation between ACE 1/ACE 2 ratio and biochemical and clinical data in the SCD group. We can attribute this finding to our cohort that had a small number of volunteers and to the fact that the patients were not in a VOC at the time.

Finally, this was a cross-sectional study with samples taken in one occasion. It is not possible to predict changes that occur over time in individual patients, and further studies are required to investigate the clinical implications of our observations.

In conclusion, our data reveal high urinary ACE 1 activity, differing from plasmatic level, in the SCD group suggesting a dissociation between the intrarenal and systemic RAAS. The increase of urinary ACE 1 activity in the SCD group suggests higher levels of Ang II with a predominance of classical RAAS axis, that can induce kidney damage. Urinary ACE 1 activity was not correlated with urine UACR, suggesting tubular damage even before glomerular injury.

Further studies are necessary to analyze other RAAS components from the alternative axis, such as enzymes like chymase, cathepsin D, neprilysin, that are able to produce Ang II and Ang 1-7, the vasoconstrictor and vasodilator peptides, respectively.
