# Erratum

**DOI:** 10.1590/2175-8239-JBN-2020-0174er

**Published:** 2021-10-18

In the article "**Levels of angiotensin-converting enzyme 1 and 2 in serum and urine of children with Sickle Cell Disease**", with DOI code number https://doi.org/10.1590/2175-8239-JBN-2020-0174, published at Brazilian Journal of Nephrology, 43 (3), 2021:

Where it was written:

We observed **lower** urinary ACE 1 activity in the SCD group versus control (p=0.005), and the SCD group and the Control Group had similar serum ACE 1 and ACE 2 activities (p 0.066; 0.058) (Table 1 and Figure 1).

Should read:

We observed **greater** urinary ACE 1 activity in the SCD group versus control (p=0.005), and the SCD group and the Control Group had similar serum ACE 1 and ACE 2 activities (p 0.066; 0.058) (Table 1 and Figure 1).

